# Adherence to the Mediterranean diet and risk of gestational diabetes: a prospective cohort study

**DOI:** 10.1186/s12884-023-05960-4

**Published:** 2023-09-08

**Authors:** Fatemeh Mohtashaminia, Fatemeh Hosseini, Ahmad Jayedi, Majid Mirmohammadkhani, Alireza Emadi, Leila Takfallah, Sakineh Shab-Bidar

**Affiliations:** 1 Student Research Committee, Semnan University of Medical Sciences, Semnan, Iran; 2 Department of Community Nutrition, School of Nutritional Science and Dietetics, Tehran University of Medical Sciences (TUMS), No 44, Hojjat-dost Alley, Naderi St., Keshavarz Blvd, Tehran, Iran; 3 Social Determinants of Health Research Center, Semnan University of Medical Sciences, Semnan, Iran; 4 Food Safety Research Center (salt), Semnan University of Medical Sciences, Semnan, Iran; 5 Department of Midwifery, Semnan Branch, Islamic Azad University, Semnan, Iran

**Keywords:** Mediterranean diet, Diet quality, Pregnancy, Gestational diabetes, Cohort

## Abstract

**Background:**

Limited data is available on the association between adherence to the Mediterranean diet during early pregnancy and risk of gestational diabetes (GDM) in countries located in the Middle East, one of the regions with the highest prevalence of GDM.

**Methods:**

A total of 647 pregnant mothers were included in the present prospective birth cohort study in Iran. Dietary intake was assessed by a 90-item food frequency questionnaire during the first trimester of pregnancy. Cases of GDM were ascertained by a two-step approach with a 50-g screen followed by a 100-g oral glucose tolerance for those who tested positive. Cox proportional hazard model was used to calculate the hazard ratio and 95%CI of GDM across tertiles of the Mediterranean diet score, while controlling for a wide range of potential confounders.

**Results:**

A total of 647 pregnant mothers were included, of whom 77 mothers were diagnosed with GDM during their pregnancy. The average age of the mothers was 28.8 ± 5.1 years. In the multivariable analysis, being in the third tertile of the score of adherence to the Mediterranean diet was associated with a 41% lower risk of developing GDM as compared to those in the first tertile (adjusted hazard ratio: 0.59, 95%CI: 0.35, 0.99).

**Conclusions:**

Based on our findings, greater adherence to the Mediterranean diet during early pregnancy may be associated with a lower risk of developing GDM in Iranian women. Larger cohort studies are needed to confirm the findings.

**Supplementary Information:**

The online version contains supplementary material available at 10.1186/s12884-023-05960-4.

## Introduction

Pregnancy is a physiological condition associated with various complications such as high blood glucose, hypertension, and preeclampsia. Gestational diabetes mellitus (GDM) is defined as impaired glucose tolerance recognized or begun for the first time during the second or third trimesters of pregnancy [1]. The global prevalence of is about 14%, with the highest prevalence being reported in the Middle East including Iran [2]. A meta-analysis of 24 observational studies which were conducted between 2002 and 2014 suggested a high prevalence of GDM in Iran, ranging from 1.3 to 18.8% [3]. GDM may lead to an increased incidence of postpartum diabetes in the mothers [4], and some other adverse maternal and fetal complications such as macrosomia, birth trauma, shoulder dystocia, and higher cesarean section rates [5] during the postpartum pregnancy period [6–9].

Studies have shown that nutrients intake and dietary patterns during pregnancy may be related to maternal and child health outcomes [10, 11]. A case-control study in Iran indicated that adherence to a dietary pattern rich in fruits, vegetables, and low-fat dairy products may be associated with a diminished risk of GDM [12]. Another prospective cohort study demonstrated that adhering to a Prudent dietary pattern in pregnancy was associated with a lower risk of GDM, especially among women with overweight or obesity [13]. In total, existing evidence suggests that increasing the consumption of vegetables, fruits, and whole grains or low consumption of ready-to-eat meals and simple sugars may be associated with a lower risk of pregnancy complications such as GDM and preeclampsia [14].

The Mediterranean dietary pattern is a healthy dietary pattern generally defined as high intake of whole grains, vegetables, fruits, nuts, seeds, and legumes, moderate intake of alcohol, and low intake of red meat, poultry, and dairy products [15]. Previous studies have indicated that greater adherence to the Mediterranean diet may be associated to a lower risk of developing GDM [16–18]. However, evidence is lacking about the association between adherence to the Mediterranean diet and risk of GDM in the Middle East. Therefore, due to the high prevalence of GDM in the Middle East including Iran and the lack of studies to investigate the potential relationship between the Mediterranean diet and GDM in Iran, the present prospective cohort study aimed to assess the relationship between adherence to Mediterranean dietary patterns during early pregnancy and the risk of GDM in a sample of Iranian women.

## Methods

### Participants

This prospective cohort study was conducted within the framework of the Persian (Prospective Epidemiological Research Studies in IRAN) Birth Cohort [19]. The Persian Birth Cohort is an ongoing, nationwide, prospective cohort study conducted in five districts of Iran to provide scientific evidence and advance knowledge for developing evidence-based national policies on different aspects of developmental origins of health and diseases [19]. This cohort study investigates the potential associations of lifestyle, environmental, and socioeconomic factors with pregnancy outcomes and mother-child mental and physical health and well-being. Participants were selected from pregnant women living in Semnan, a city located in central Iran. During 2018–2020, pregnant women who were referred to health care centers in Semnan were invited to participate in this prospective cohort study. In addition, we used advertisements through local and social media and medical clinics throughout the city to encourage women to participate in this prospective cohort study. Inclusion criteria were women of Iranian nationality who were within the first trimester of pregnancy, irrespective of parity or use of fertility treatment, who have resided in Semnan for at least one year and plan to give birth in a hospital located in Semnan and did not have a history of diabetes, cancer or cardiovascular disease. Pregnancies ending in either natural vaginal delivery or cesarean section were included. Exclusion criteria were having twin gestations and hormone-related diseases or hormone therapy.

In total, 1024 women agreed to participate in the study. Of those, mothers who did not complete dietary questionnaires during the first trimester (n = 281), those who did not continue the study until the end and had incomplete information about study outcomes (n = 45), mothers with total energy intake outside the range of 800 to 4200 kcal/day (n = 18), those who used cigarette smoking (n = 10), and mothers with a history of GDM in previous pregnancies (n = 23) were excluded from the analyses, leaving 647 pregnant women for the present study (Supplementary Fig. 1). The protocol of the study was explained to all participants, and all participants provided a signed informed consent form. All methods were carried out in accordance with guidance outlined in Declaration of Helsinki. The ethic committee of the Semnan University of Medical Sciences approved the study protocol (Ethic code: IR.SEMUMS.REC.1400.201).

### Assessment of dietary intake

Dietary intakes of the participants during the first trimester of pregnancy were evaluated by using a 90-item food frequency questionnaire that was developed and validated for use in this prospective cohort study [19, 20]. Dietary assessments were performed through face-to-face interviewing using trained interviewers. We asked mothers to report their frequency of consumption of listed food items in the FFQ, based on commonly used units or portion sizes, over their first trimester of pregnancy. The frequency response categories were nine multiple-choice categories varying from “never or less than once a month” to “6 or more times per day” depending on the nature of food items. All reported consumption frequencies were converted to grams per day using household measures. We used Nutritionist IV software (version 7.0; N-Squared Computing, Salem, OR) [21], modified for Iranian foods [22], to calculate the total energy and nutrient intakes.

### Calculation of Mediterranean diet pattern

The Mediterranean dietary pattern adherence score was calculated by including eight food groups including (1) fruits, (2) vegetables, (3) nuts, (4) legumes, (5) fish, (6) ratio of monounsaturated to saturated fats, (7) meats including processed and unprocessed red meat and poultry, and (8) dairy products. To calculate the score of adherence to the Mediterranean diet pattern, intake of each food group was categorized into two groups including less and more than the median. We assigned the first six groups one point for being in the upper median category and zero point for being in the lower than the median category. In contrast, for groups seven and eight, we used an inverse scoring system. We summed the scores of all eight food groups and then, categorized participants into tertiles based on their total Mediterranean dietary pattern score (ranging from 0 to 8) [15]. Because intake of whole grains is very small in the traditional Iranian diet [23], we did not include whole grains when calculating the score of adherence to the Mediterranean diet. Therefore, the range of the calculated Mediterranean diet score was from 0 to 8, with higher scores indicating greater adherence to the Mediterranean diet.

### Outcome assessment

GDM was defines as increased blood glucose levels and symptoms of diabetes in pregnant women who has not previously been diagnosed with diabetes. Having at least two of the following criteria, based on a two-step approach with a 50-g (nonfasting) screen followed by a 100-g oral glucose tolerance test for those who screen positive, was considered diagnosis of GDM: fasting blood sugar higher than 95 mg/dL, one-hour blood sugar higher than 180 mg/dL, two-hour blood sugar higher than 155 mg/dL, three blood sugar hours greater than 140 mg/dL and/or pharmacological treatment of GDM [24]. In case of mothers who used pharmacological treatments for GDM, medical records and laboratory measurements were checked to confirm diagnosis of GDM.

### Assessment of other variables

Information about the characteristics of the study participants were obtained by trained interviewers using structured pre-tested questionnaires that were developed for using in Persian Birth Cohorts [19]. Information about age, history of diseases, educational level, mother- and father’s occupational status, and family income were recorded by trained interviewers. We used the generally validated International Physical Activity Questionnaire (IPAQ) to evaluate physical activity levels [25]. Based on Metabolic Equivalents minutes per week (MET-min/week) [26], participants were grouped into two categories no or low physical activity (< 3000 MET-minute/week) and moderate and high low physical activity (> 3000 MET-minute/week). Weight and height were measured by a trained interviewer. Weight was measured at the study baseline using a digital scale to the nearest 0.5 kg with light clothes and without shoes. Weight measurement was repeated in the second and third trimesters. The final weight of the mothers was measured using the same protocol in the hospital before delivery. Weight gain was calculated by subtracting the first weight from the last weight. Height was measured in a standing position with a tab measured to the nearest 0.5 centimeter by asking the participants to stand without shoes and shoulders touching the wall. Body mass index (BMI) was calculated based on the weight in kilograms divided by height in meters squared.

### Statistical analyses

First, we calculated the score of adherences to the Mediterranean diet pattern and then, grouped study participants across tertiles of the Mediterranean diet pattern score. Secondly, we presented characteristics of the study participants across tertiles of the Mediterranean diet pattern. We used ANOVA test for continuous variables and χ2 test for categorical variables to compare participants’ characteristics across categories of the Mediterranean diet. We used Cox proportional hazard model to calculate hazard ratio (HR) and 95% confidence interval (CI) of GDM across categories of the Mediterranean diet. We first calculated crude HRs and 95%CIs of GDM across categories of the Mediterranean diet score. Then, we performed multivariable analyses by controlling for potential confounders including maternal age, mother’s job and educational levels (Illiterate, under diploma, diploma, university graduate), physical activity (no or low/moderate to high), father’s income, BMI before pregnancy, history of hypertension before pregnancy (yes/no), history of hyperthyroidism and hypothyroidism before pregnancy (yes/no), weight gain during current pregnancy (kg), and total energy intake (kcal/d). Of these confounders, age, physical activity, total energy intake, and socioeconomic status (we included mother’s job and education and father’s income as proxy of socioeconomic status) as general confounders that should be included in the analyses. Weight gain during pregnancy [27] and pre-pregnancy BMI [28] are also the two important risk factor for GDM. In addition, there is evidence that thyroid disorders before pregnancy [29] and history of hypertensive disorders [30] are associated with the risk of GDM. Therefore, we included these variables as confounders in the analyses. Information about potential confounders were obtained when recruiting the participants at the baseline of the study. All statistical analyses were carried out using SPSS (SPSS Inc., version 22). P values were considered significant at < 0·05.

## Results

Table 1 shows the characteristics of the study participants (n = 647) across categories of the Mediterranean diet adherence score. Mothers in the second tertile of the Mediterranean diet score were more likely to have nausea during current pregnancy, and lower number of previous pregnancies. There is no other significant difference in terms of general characteristics of the mothers across tertiles of the Mediterranean diet score.

**Table 1:** Characteristics of the study participants across categories of the Mediterranean diet adherence score (n = 647)

Variable	Tertile 1	Tertile 2	Tertile 3	P^*^
Age (years)	28.3 ± 5.1	28.9 ± 5.4	29.2 ± 4.8	0.05
Prepregnancy BMI (kg/m^2^)	25.3 ± 4.4	24.8 ± 3.8	25.2 ± 4.9	0.81
Weight gain during current pregnancy (kg)	13.7 ± 5.3	13.2 ± 5.3	13.5 ± 4.6	0.76
Having job with income (%)	40.6	20.3	39.0	0.05
University graduate (%)	35.5	26.7	37.7	0.74
Physical activity				
Low (%)	35.5	24.7	39.8	0.60
Moderate (%)	36.9	32.5	30.6	0.60
History of CVD (%)	42.9	14.3	42.9	0.73
History of hypertension (%)	75.0	25.0	0	0.11
History of hypothyroidism (%)	35.3	25.0	39.7	0.85
History of hyperthyroidism (%)	44.4	33.3	22.2	0.61
History of pregnancy hypertension (%)	45.5	9.1(%)	45.5	0.11
Order of pregnancy (≥ 3, %)	35.9	26.6	37.5	0.03
Nausea during current pregnancy (%)	40.3	23.6	36.1	0.04
Multivitamin use during current pregnancy (%)	31.0	28.2	40.80	0.65

^*^ Obtained by ANOVA test for continuous variables and chi-square test for categorical variables.

Abbreviations: BMI, body mass index; CVD, cardiovascular disease.

Table 2 shows the intake of micro- and macronutrients and food groups across tertiles of the Mediterranean diet score. In our study population, a higher Mediterranean diet score was related to a higher intake of total fat, total protein, saturated fat, monounsaturated fatty acids (MUFAs), dietary fiber, vitamin C, magnesium, and calcium. Also, Table 2 shows the food group intakes based on tertiles of the Mediterranean diet score, where a higher score was related to a higher intake of fruit, vegetable, diary intake, legumes, nuts, poultry and egg.

**Table 2:** Dietary intake of the study participants across categories of the Mediterranean diet adherence score (n = 647)

Variable	Tertile 1	Tertile 2	Tertile 3	P^*^
Energy (kcal/d)	1579 ± 563	1658 ± 235	1758 ± 365	0.23
Nutrients				
Carbohydrate (g/d)	153 ± 45	178 ± 29	159 ± 56	0.12
Total fat (g/d)	62.97 ± 20.1	67.24 ± 22.6	71.19 ± 22.7	< 0.001
Total protein (g/d)	47.50 ± 20.9	53.82 ± 20.6	62.45 ± 24.1	< 0.001
Saturated fat (g/d)	18.27 ± 6.1	19.40 ± 5.8	20.49 ± 6.8	< 0.001
PUFA (g/d)	20.33 ± 7.8	20.84 ± 8.6	20.94 ± 8.1	0.40
MUFA (g/d)	14.52 ± 4.7	15.67 ± 4.1	17.13 ± 5.4	< 0.001
Dietary fiber (g/d)	11.68 ± 4.7	14.57 ± 4.8	18.79 ± 6.8	< 0.001
Vitamin C (mg/d)	186.60 ± 101.0	235.93 ± 94.4	333.03 ± 214.7	< 0.001
Magnesium (mg/d)	211.13 ± 74.8	243.81 ± 74.1	301.31 ± 113.3	< 0.001
Calcium (mg/d)	704.09 ± 332.9	774.13 ± 329.9	922.08 ± 450.8	< 0.001
Food groups				
Grains (g/d)	20.45 ± 23.4	22.82 ± 25.8	24.53 ± 24.1	….
Dairy (g/d)	317.35 ± 208.7	337.71 ± 210.2	398.49 ± 285.9	< 0.001
Fruits (g/d)	270.53 ± 195.6	352.01 ± 189.7	498.35 ± 259.5	< 0.001
Vegetables (g/d)	193.66 ± 99.1	249.77 ± 103.9	325.57 ± 136.1	< 0.001
Legumes and nuts (g/d)	11.62 ± 9.6	17.74 ± 13.3	25.01 ± 11.6	< 0.001
Red and processed meat (g/d)	13.26 ± 10.1	12.85 ± 9.1	14.15 ± 11.5	0.34
Poultry (g/d)	8.15 ± 7.9	10.45 ± 10.4	10.62 ± 9.9	0.004
Egg (g/d)	20.12 ± 19.9	22.32 ± 28.8	24.73 ± 18.6	0.02

^*^ Obtained by ANOVA test.

Abbreviations: PUFAs, Polyunsaturated fatty acids; MUFAs, Monounsaturated fatty acids.

Table 3 indicates the HR and 95% CI of GDM (n = 77 cases) across tertiles of the Mediterranean diet score. In the crude model, there was no association between adherence to the Mediterranean diet score and risk of GDM. However, higher score of adherence to the Mediterranean dietary pattern during first trimester of pregnancy was significantly related to lower risk of developing GDM after controlling for maternal age, education, marital status, having job with income, pre-pregnancy BMI, order of pregnancy, nausea during current pregnancy, multivitamin use during current pregnancy, physical activities, total energy intake and history hypertension, hypothyroidism, hyperthyroidism (adjusted HR: 0.59, 95%CI: 0.35, 0.99) (Table 3).

**Table 3:** Hazard ratio (95% CI) of gestational diabetes by tertiles of the Mediterranean diet adherence score (n = 647)

Tertile	Tertile 1	Tertile 2	Tertile 3
MED score	0–3	3–6	6–8
Participants	230	173	244
Person-week	23,802	6547	9096
Cases	37	12	28
HR and 95%CI			
Crude	1	1.08 (0.66, 1.77)	0.73 (0.44, 1.20)
Multivariable adjusted*	1	0.91 (0.55, 1.51)	0.59 (0.35, 0.99)
P-value	-	0.23	0.01

*Obtained by Cox proportional hazard regression model, and adjusted for maternal age, physical activity, mother’s job and education, father’s income, pre-pregnancy body mass index, weight gain during current pregnancy, total energy intake, and history of hypertension, hypothyroidism, and hyperthyroidism

## Discussion

The main finding of the present prospective study showed that greater adherence to a modified Mediterranean diet during early pregnancy may be associated with a lower risk of GDM among Iranian women. Although previous studies have suggested that socioeconomic factors such as occupation, education, and family income may be important in determining individuals’ food choices, but among our participants, there was no significant difference in terms of the general characteristics of the mothers across tertiles of the Mediterranean diet score.

Existing evidence suggests that maternal diet quality either during or before pregnancy may have potential effects on the health status of the mother and infants. A prospective evaluation within the Nurse’s Health Study II in the US suggested that greater score of the Alternative Healthy Eating Index-2010 before pregnancy might be associated with a reduced risk of GDM [31]. In a prospective cohort study in 10 Mediterranean countries, greater adherence to the Mediterranean dietary pattern was associated with a lower incidence of GDM and better degree of glucose tolerance, even in women without GDM [17]. Two other prospective observational studies in Iran [16] and Greece [17] also found an inverse association between Mediterranean diet adherence score during pregnancy and likelihood of GDM. Also, greater adherence to the Mediterranean diet was inversely associated to the risk of type 2 diabetes, metabolic syndrome, and premature death in the general population [32–37].

With regards to intervention studies, a randomized trial conducted in 874 Spanish pregnant women indicated that dietary intervention with a Mediterranean diet supplemented with extra virgin olive oil and pistachio during early pregnancy can reduce the incidence of GDM as compared to a standard fat-restricted diet [38]. However, a meta-analysis of five randomized trials indicated that dietary interventions with a healthy diet during pregnancy did not reduce in the incidence of GDM [39].

The Mediterranean diet is a healthy plant-based dietary pattern rich in fruits and vegetables, vegetable oils, complex carbohydrates, dietary fibers, calcium, magnesium, potassium, vitamin C and phytochemicals [40]. Evidence from observational studies suggests a potential link between dietary magnesium intake and risk of type 2 diabetes [41], a finding that confirmed by that of randomized trials indicating effectiveness of magnesium supplementation in improving insulin resistance and glycemia [41]. Higher intake of different types of dietary fibers and phytochemicals derived from fruit and vegetables was also associated with improved insulin sensitivity and lower risk of developing type 2 diabetes [42, 43]. Lower dietary glycemic index due to the high consumption of complex carbohydrates may be another potential pathways mediating the link between the Mediterranean diet and GDM [44]. Intervention studies also indicated that adherence to the Mediterranean diet can ameliorate oxidative stress, inflammation, and endothelial dysfunction [45–47]; the three important underlying mechanisms implicated in developing insulin resistance and GDM [48]. In the present study, we used a modified version of the Mediterranean diet score which did not include information about whole grain intake. However, our findings suggested that a greater adherence to a Mediterranean-style dietary pattern, even without whole grain intake, could be associated with a lower risk of GDM in Iranian mothers.

The strengths of the study are its population-based prospective observational setting, using pre-defined and validated questionnaires and trained interviewers to obtain the data, and controlling for a wide range of confounders. In addition, most of the evidence regarding the association of dietary patterns and risk of GDM is from Western countries, and limited data is available from countries located in the Middle East. The prevalence of GDM in the Middle East is about 28%, as compared to average prevalence of 14% across the globe [2]. The highest global prevalence of GDM in the Middle East indicates that there is a pressing need to focus on lifestyle modifications in order to reduce the incidence of GDM. Our findings indicated that greater adherence to the Mediterranean diet in countries located far from regions wherein the traditional Mediterranean diet was originated can present important benefits in favor of lowering the risk of GDM and thus, may have important public health implications. There are also some limitations for consideration. We reported the results of an observational study and thus, our findings are subject to bias due to residual confounding by unmeasured or unrecorded confounders. We used a food frequency questionnaire for dietary assessment that is subject to measurement error [49]. In addition, we performed dietary assessment during the first trimester and thus, did consider potential changes in the quality of the diet during pregnancy. Finally, we used a modified version of the Mediterranean diet score which did not include whole grains. Therefore, more prospective cohort studies are needed to investigate the association between adherence to the Mediterranean dietary pattern during early pregnancy and risk of developing GDM considering all components of the original Mediterranean diet.

## Conclusions

Our results suggest that greater adherence to the Mediterranean diet during early pregnancy may be associated with a lower risk of developing GDM. Larger prospective cohort studies are still needed to confirm our findings.

### Electronic supplementary material

Below is the link to the electronic supplementary material.

Supplementary Material 1

## Data Availability

Data described in the manuscript, code book, and analytic code will be made available upon request to the corresponding author.
