# Supplementary figures and images for: Adherence to the Mediterranean diet and risk of gestational diabetes: a prospective cohort study
Source: BMC Pregnancy Childbirth. 2023 Sep 8;23:647. doi: 10.1186/s12884-023-05960-4 (PMC10486001; doi:10.1186/s12884-023-05960-4)

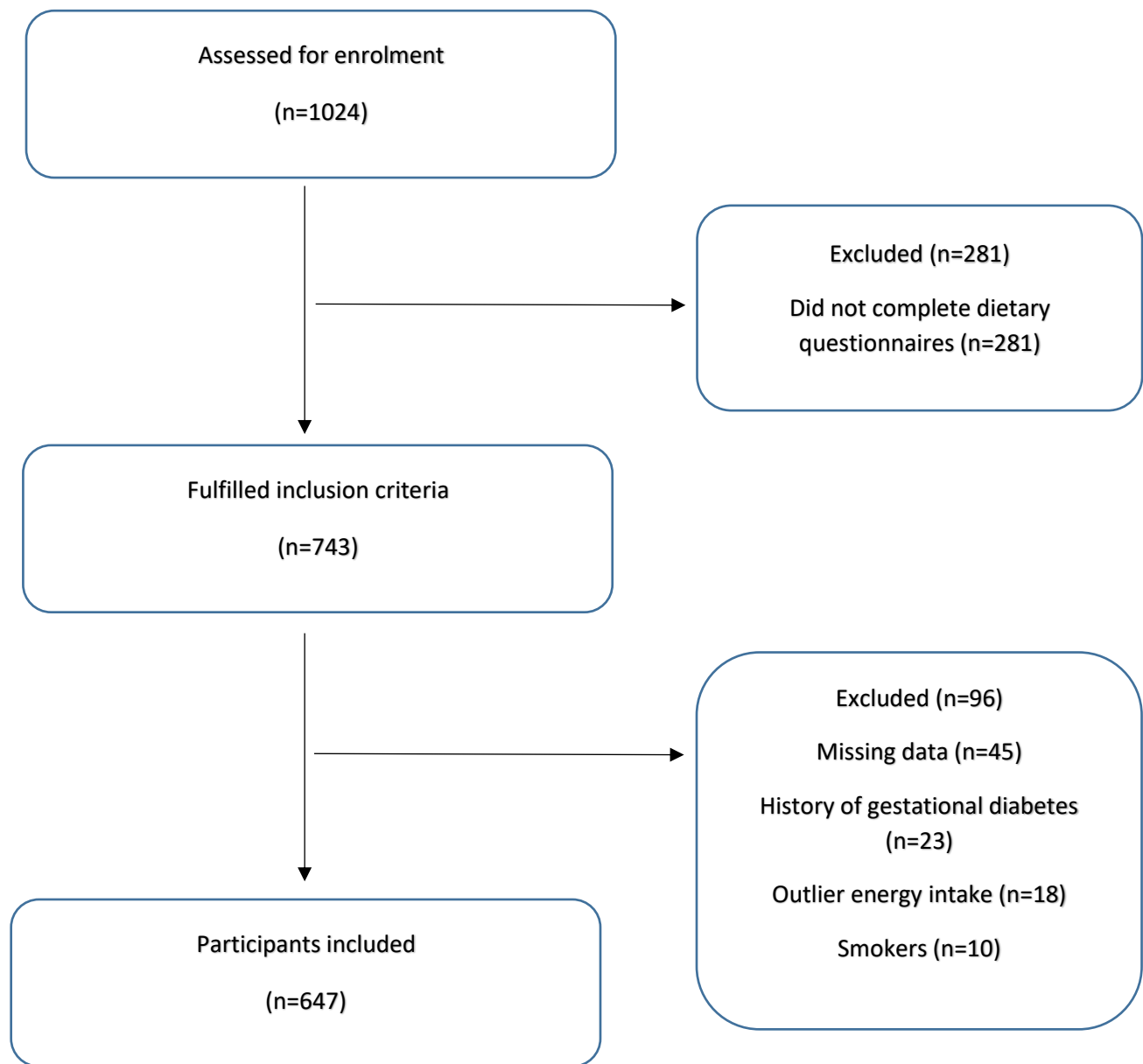

**Supplementary Figure 1.** Flowchart of the study.

Supplement: Supplementary file 1 — Supplementary Material 1 [file 12884_2023_5960_MOESM1_ESM.pdf]
